# A Comparative Study on Minimal Flow Anaesthesia in Geriatric and Middle-aged Patients

**DOI:** 10.4274/TJAR.2025.241740

**Published:** 2025-07-24

**Authors:** Sinan Ünsal, Gülay Erdoğan Kayhan, Meryem Onay, Mehmet Sacit Güleç

**Affiliations:** 1 University of Health Sciences Türkiye, Gaziantep City Hospital, Clinic of Anaesthesiology and Reanimation, Gaziantep, Türkiye; 2 Eskişehir Osmangazi University Faculty of Medicine, Department of Anaesthesiology and Reanimation, Eskişehir, Türkiye

**Keywords:** Anaesthesia, geriatrics, intraoperative awareness, oxygen consumption, rebreathing

## Abstract

**Objective:**

Minimal flow anaesthesia reduces costs and environmental pollution, and has a protective effect on the respiratory tract. This study aimed to compare the ease and tolerability of minimal flow anaesthesia in the geriatric and middle-aged patient populations.

**Methods:**

In this prospective study, we enrolled 40 patients between 18 and 50 years (Group Y) and 40 patients 65 years or older (Group E), scheduled for abdominal surgery under general anaesthesia. Following a period of high flow with desflurane in O_2_/air, the fresh gas flow was reduced to 350 mL min^-1^. Desflurane concentration was adjusted to maintain a bispectral index between 40 and 50. The oxygen concentration in fresh gas flow was titrated by ±10%. Throughout the surgery, gas concentrations, oxygenation parameters, hemodynamic data, and the depth of anaesthesia were monitored. The number of alterations in fresh gas oxygen and desflurane concentrations was recorded.

**Results:**

The depth of anaesthesia and oxygenation parameters were adequately sustained within safe limits among all patients, while the number of changes in the fresh gas flow oxygen levels was found to be significantly lower in geriatric patients. The increase in the number of oxygen level was 1.1±0.8 in Group E and 1.8±1.2 in Group Y (*P*=0.006). Total alteration in oxygen was 1.2±1 in Group E and 1.9±1.3 in Group Y (*P*=0.01). Oxygenation parameters consistently remained within clinically acceptable ranges in both groups, and the amount of change in desflurane concentration showed no intergroup difference.

**Conclusion:**

Administering minimal flow anaesthesia at a rate of 350 mL min^-1^ in the geriatric population, compared to the younger population, can be performed requiring less manipulation, without inducing hypoxia or inadvertent awareness.

**Main Points**

• Minimal flow anaesthesia (MFA) reduces costs and environmental pollution and has a protective effect on the respiratory tract.

• The incorporation of MFA as an anaesthesia modality should be promoted.

• To the best of our knowledge, this is the first study of comparing the impacts of MFA across various age demographics.

• MFA can be performed without inducing hypoxia or inadvertent awareness in suitable elderly patients.

## Introduction

The re-breathing of ≥50% of exhaled air within the semi-closed re-breathing circuits present in contemporary anaesthesia workstations is commonly denoted as low flow anaesthesia (LFA). According to the sub-classification of flow rates of gases into anaesthetic circuits proposed by Baker^1^ and Simionescu, very high flow is defined as >4 L min^-1^, high flow as 2-4 L min^-1^, medium flow as 1-2 L min^-1^, low flow as 0.5-1.0 L min^-1^, minimal flow as 0.25-0.5 L min^-1^, and metabolic flow as <0.25 L min^-1^.^1^ LFA offers several pivotal advantages, including cost reduction, decreased environmental pollution, respiratory tract protection, and the preservation of body temperature. Therefore, it is recommended to reduce the fresh gas flow (FGF) in the anaesthesia machines.^2^

The safe administration of LFA requires close monitoring by an anaesthesiologist and the use of technical equipment available in all modern anaesthesia machines. This enables monitoring of inspiratory and expiratory gas concentrations (oxygen, carbon dioxide, inhalation anaesthetics), as well as minute volume (MV) measurements. When necessary precautions are not taken, potential risks associated with LFA applications include hypoxia and inadequate depth of anaesthesia. Preventing hypoxia and ensuring an adequate level of anaesthesia necessitate the establishment of alarm thresholds aligned with predetermined targets. In instances where these thresholds are exceeded, adjustments to oxygen and inhalation anaesthetic levels become imperative. Such adjustments, accompanied by meticulous monitoring, play a pivotal role in maintaining patient safety during LFA administration.

Anaesthetic oxygen consumption during anaesthesia is estimated using the Brody formula [10 x bodyweight kg^3/4^], with the outcome varying according to weight. However, it is suggested that due to decreased muscle mass and slowed metabolism in elderly patients, their oxygen requirement during surgery might be lower. A recent study conducted on geriatric patients undergoing major abdominal surgery suggested that their oxygen requirement decreased to one-third.^3^ Additionally, the minimum alveolar concentration (MAC) determining the requirement for inhalation anaesthetics decreases by 6% per decade, and it is well-known that MAC values are lower in elderly patients.^4^

We hypothesized that minimal flow anaesthesia (MFA) at 350 mL min^-1^ would be manageable and tolerated in older adult patients, given the presumed decreased requirement for oxygen and inhalation anaesthetics. To investigate this hypothesis, we compared young, middle-aged, and older adult patients undergoing elective open abdominal surgery under general anaesthesia with MFA. The primary comparison focused on the number of interventions in oxygen and inhalation anaesthetic levels, alongside evaluations of oxygenation parameters and depth of anaesthesia.

## Methods

This prospective study was conducted following approval from the Eskişehir Osmangazi University, Clinical Trials Ethics Committee of Studies (approval no.: 21, dated: December 22, 2022), and informed consent from all patients was obtained. The study included patients undergoing elective open abdominal surgery lasting more than 2 hours under general anaesthesia, in the supine position, and classified as American Society of Anesthesiologists physical status I-III. A total of 80 patients, comprising 40 elderly patients aged 65 and above (Group E), and 40 younger patients aged between 18-50 (Group Y), were planned to be enrolled in the study. Patients were excluded from the study if they had severe cardiovascular and respiratory diseases, cerebrovascular diseases, uncontrolled diabetes, profound anaemia (hemoglobin <7 g dL^-1^), a body mass index (BMI) of <20 or >35, were heavy smokers (>1 pack/day), had alcohol or drug addiction disorder, had expected increases in O_2_ consumption (such as in sepsis or hyperthermia), or did not agree to participate in the study.

Patients admitted to the operating room without premedication underwent monitoring procedures, including electrocardiography, peripheral oxygen saturation (SpO_2_), non-invasive blood pressure measurements, bispectral index (BIS) monitoring using Datex-Ohmeda equipment, and near-infrared spectroscopy (NIRS) (COVIDIEN Invos 5100C Somanetics, Massachusetts, USA) for regional cerebral oxygen saturation on the right (rSO_2_-R) and left (rSO_2_-L) hemispheres. Additionally, neuromuscular monitoring was conducted using the ulnar nerve and adductor pollicis muscle. Baseline values were recorded prior to induction.

The Dräger Perseus^® ^A500 anaesthesia machine (Dräger Medical, Lubeck, Germany) was employed in this study. Prior to anaesthesia induction, the soda lime was replaced for all patients, and the anaesthesia apparatus and breathing circuit underwent an automated test. The anaesthesia machine’s alarms were set as follows: a lower limit of 30% for inspiratory oxygen concentration (fiO_2_), an upper limit of 5 mmHg for inspiratory carbon dioxide concentration (fiCO_2_), 30-45 mmHg for end-tidal carbon dioxide pressure (etCO_2_), 5-30 cmH_2_O for peak pressure (P_peak_), and an expiration gas volume lower limit set 500 mL below the desired MV. If FiCO_2_ exceeded by 5 mmHg, the CO_2_ absorber (Drägersorb) was to be replaced.

All patients were pre-oxygenated with 100% O_2 _for 3 minutes. For induction of anaesthesia, 4-7 mg kg^-1 ^thiopental, 1 μg kg^-1^ fentanyl, and 0.6 mg kg^-1^ rocuronium bromide were administered. After induction, radial artery cannulation was performed in all patients, followed by invasive artery monitoring. After achieving adequate muscle relaxation [train of four (TOF): 0], endotracheal intubation was performed. Ventilation was maintained with volume auto-flow (AF) mode with 30-45 mmHg EtCO_2_ (tidal volume, 6-8 mL kg^-1^; positive end expiratory pressure, 5 cmH_2_O; and respiratory frequency 10-14 min). Anaesthesia maintenance started in both groups with a FGF of 4 L min^-1^, consisting of a mixture of 50% oxygen and 50% air and 6-8% desflurane. When the BIS value reached a value between 40 and 50 (approximately 10 minutes later), the total gas flow was set to 350 mL min^-1^, with an oxygen concentration (fgO_2_) of 70% and a desflurane concentration (fgDes) of 12% within the FGF. Throughout the maintenance of anaesthesia, the desflurane concentration was titrated, and reduced to maintain the BIS value within the range of 40 to 50. Concurrent with surgical incision, all patients received a remifentanil infusion (20 μg mL^-1^) at a rate of 0.08-0.1 μg kg^-1^ min^-1^.

Throughout the surgical procedure, it was planned to increase the fgO_2_ by 10% (80%, 90%, 100%) under specific conditions: if the fiO_2_ dropped below 30%, if SpO_2_ decreased to <95%, or in the event of a 20% reduction in cerebral oximetry values (rSO_2_-L or rSO_2_-R) compared to baseline. If any of these parameters fell below the specified targets despite the fgO_2_ being set at 100%, the anaesthesiologist planned to increase the FGF to 500 mL min^-1^. If necessary, they intend to further increase fgO_2_ by 10% until reaching the target values.

Throughout the operation, FGF rate, and gas components (fgO_2_, fgDes), inspiratory and expiratory gas concentrations [desflurane (fiDes and etDes), O_2_, and CO_2_ values], rSO_2_-L and rSO_2_-R, hemodynamic data, TOF, and BIS levels were recorded. After induction, recordings were taken every 5 minutes for the initial 30 minutes and subsequently every 10 minutes for the following 90 minutes.

Total interventions (reductions and increases) in fgO_2_ and fgDes levels were noted at the end of two hours. Arterial blood gas samples were obtained from all patients at 10 and 70 minutes, and the results (pH, pO_2_, pCO_2_, lactate, and HCO_3_) were recorded. Data recording terminated at the end of the second hour, and gas consumption was noted. Patients were visited 24 hours postoperative and queried regarding intraoperative awareness using the modified Brice scale (“What is the last thing you remember before going to sleep?”; “What is the first thing you remember after waking up?”; “Did you have any dreams or other experiences while sleeping?”).^5^

When mean arterial pressure (MAP) increased by 20% or more from baseline for 1 minute or longer, the remifentanil infusion was increased. In the absence of a satisfactory response, intravenous nitroglycerin was planned, in pulse or infusion form. If the MAP dropped below 65 mmHg for a duration of 1 minute or longer, the primary intervention involved intravenous crystalloid fluid replacement, with subsequent administration of 5-10 mg of ephedrine in case of persistent hypotension. Atropine administration was scheduled at a heart rate (HR) below 45 beats per minute (bpm). If the HR exceeded 100 bpm, an initial 20 μg bolus of remifentanil was planned, followed by potential treatment with esmolol if the elevated HR persisted.

### Statistical Analysis

The sample size of the study was calculated using G*Power 3.1.9 (G*Power, Universität Düsseldorf, Germany). The primary outcome of the study was the number of interventions (reductions and increases) in fgO_2_ and fgDes levels during the first two hours of operation. From an earlier study,^6^ power analysis showed that 37 patients were necessary to detect a difference of 0.20 between the number of interventions (effect size 0.66, 5% type I error rate, 80% power, two tailed t-test). Considering study withdrawals or protocol violation, we set a sample size of 40 in each arm.

All analyses were performed using IBM SPSS Statistics version 25 (IBM Corp., Armonk, NY, USA) software package. Normality of continuous variables across groups was assessed using the Shapiro-Wilk normality test. The Independent Samples t-test and the Mann-Whitney U test were employed for between-group comparisons of continuous variables. Repeated measures analysis of variance (ANOVA) was utilized for continuous variables involving repeated measurements, with multiple comparisons assessed using the Sidak test. Group comparisons of categorical variables were conducted using the chi-square test. Various test methods such as Pearson’s chi-square, Yates’ correction, Fisher’s exact test, and Monte Carlo simulation were employed in chi-square analyses. Pearson correlation analysis was employed to determine relationships between variables. A significance level of *P *< 0.05 was set.

## Results

The study included a total of 80 patients (40 in Group E and 40 in Group Y). The demographic data of the patients are presented in Table 1.

**Figure 1:**
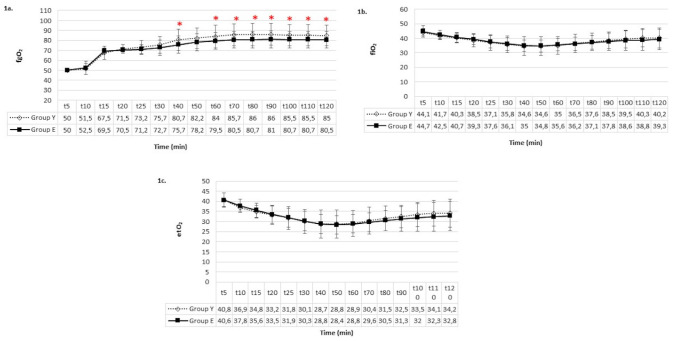
Oxygen concentrations a) fgO_2_ b) fiO_2_ c) etO_2_. *There is a significant difference between groups (*P* < 0.05). fgO_2_, fresh gas oxygen concentration; fiO_2_,inspiratory oxygen concentration; etO_2_, expiratory oxygen concentration.

The number of interventions (changes in intraoperative fgO_2_ and fgDes levels) is demonstrated in Table 2. Statistical analysis of the groups revealed a higher frequency of fgO_2_ increases and overall changes in Group Y compared to Group E (*P *< 0.05). The fgO_2_ was higher in the young patient group recorded at the 40^th^ minute and from the 60^th^ minute onward (Figure 1). Three patients in Group Y did not achieve oxygenation targets, necessitating an increase in FGF, whereas in all geriatric patients, maintaining a 350 mL min^-1^ FGF demonstrated the ability to sustain oxygenation targets with fewer interventions and lower fgO_2_ levels.

**Figure 2:**
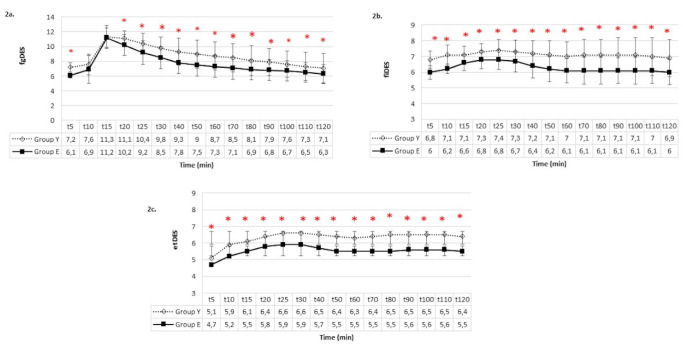
Desflurane concentrations a) fgDes b) fiDes c) etDes. *There is a significant difference between groups (*P* < 0.05). fgDes, fresh gas desflurane concentration; fiDes, inspiratory desflurane concentration; etDes, expiratory desflurane concentration.

**Figure 3:**
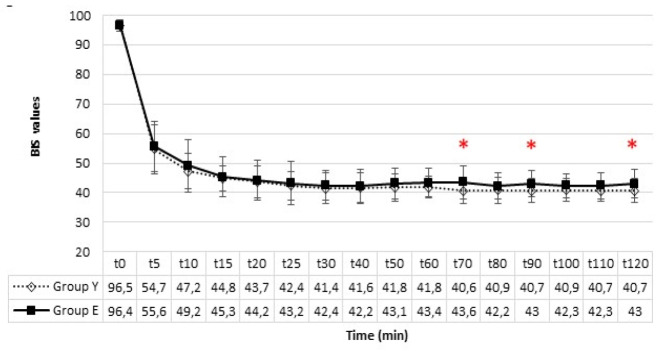
BIS values. *There is a significant difference between groups (*P* < 0.05). BIS, bispectral index.

No statistically significant difference was found between the two groups regarding interventions at desflurane levels. The fgDes, fiDes, and etDes concentrations are shown in Figure 2. A significant intergroup difference was found in terms of fgDes at all times except for the 10^th^ and 15^th^ minutes (*P *< 0.05). Group E exhibited lower desflurane concentrations overall. A statistically significant difference between groups was observed in terms of fiDes and etDes across all time points (*P *< 0.05). Group E consistently demonstrated lower fiDes and etDes levels. Throughout the operation, BIS values were maintained within the range of 40-50 (Figure 3). In the intergroup comparison of intraoperative BIS values, no significant difference was observed except minutes 70, 90, and 120, though considered clinically insignificant. Postoperative evaluation at the 24^th^ hour, did not reveal any instances of intraoperative awareness under anaesthesia in any of the patients based on the interrogation conducted.

In the intergroup comparison of intraoperative SpO_2 _values, no significant difference was observed except for minutes 0 and 5, and the difference was considered clinically insignificant.

**Figure 4:**
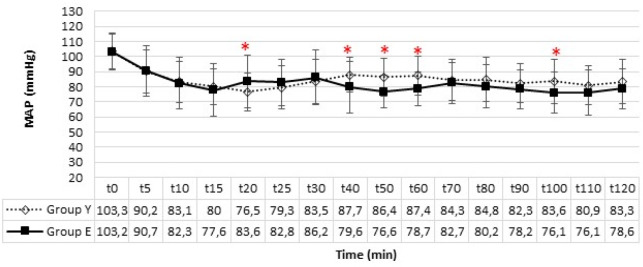
Intraoperative mean arterial pressure. *There is a significant difference between groups (*P* < 0.05). MAP, mean arterial pressure.

The rSO_2 _values obtained by cerebral oximetry in both groups were analysed. In the intergroup evaluation, a significant difference was observed in the rSO_2_-R and rSO_2_-L values at 60, 80, 90, 100, 110, and 120 minutes (*P *< 0.05) (Table 3). rSO_2_-R and rSO_2_-L were higher in Group Y; however, no patient experienced a reduction of more than 20% from the baseline levels in intragroup comparisons. There were no statistically significant differences in MAP in intragroup comparison after transitioning to MFA. In intergroup comparison, MAP was higher in Group E at the 20^th^ minute while it was lower at the 40^th^, 50^th^, 60^th^ and 100^th^ minutes (Figure 4).

Intraoperative arterial blood gas results obtained from all patients at 10 and 70 minutes are presented in Table 4. No statistically significant differences were observed between groups concerning pH, PO_2_, PCO_2_, lactate, and HCO_3 _(*P *> 0.05).

The relationships of the number of changes in fgDes and fgO_2_ with weight, body surface area (BSA), and BMI were also evaluated. A positive correlation was observed among BSA, BMI, and weight with the amount of change in O_2_ value, and the total change in all patients and separately in both groups (*P *< 0.05).

The desflurane, O_2_, air, and remifentanil consumption at the end of two hours were analysed. The desflurane consumption was significantly higher in Group Y (*P *< 0.05) (Table 5). In Group E, 19 patients received ephedrine and 3 patients received atropine. In Group Y, 18 patients received ephedrine and 3 patients received atropine.

## Discussion

As per our hypothesis, the changes in oxygen levels, reflecting the requirement for intervention during MFA, were notably reduced among geriatric patients compared to younger and middle-aged adults. Meanwhile, oxygenation parameters were consistently maintained within safe thresholds. The alteration in desflurane levels exhibited parallel patterns between the two groups, and none of the patients reported experiencing awareness during the procedure.

Due to the rising concerns regarding healthcare costs, environmental pollution, and global warming, MFA-as an anaesthesia technique-has become increasingly important in recent years due to its beneficial effects. These advantages encompass the reduction in consumption of inhalation anaesthetics and the promotion of environmental sustainability by reducing the carbon footprint.^7, 8, 9^

The evolution of technology integrated into anaesthesia machines enables the reduction of FGF by elevating the rate of rebreathing during general anaesthesia and allows for closer monitoring of adverse events such as hypoxia or intraoperative awareness under anaesthesia. Furthermore, the low solubility and metabolic rate of contemporary inhalation agents have played a crucial role in the widespread adoption of this technique.

The increasing life expectancy and improvements in analysis of diseases have resulted in the increased frequency of geriatric individuals in health services. Some of the physiologic changes that occur in the geriatric population have a negative impact on life, whereas others may compensate for these changes. Furthermore, aside from reduced metabolism and HR, a more sedentary lifestyle contributes to decreased oxygen consumption in older adults.^10^ Additionally, the concentrations of inhalation agents required for the maintenance of general anaesthesia decrease with advancing age.^4^ The current study was designed to evaluate the manageability and tolerability of MFA in older and young-middle-aged patients using a Dräger-Perseus^®^ A500 anaesthesia workstation with suitable conditions for the administration of MFA. Desflurane, which rapidly reaches equilibrium between alveolar and brain concentrations due to its low blood/gas solubility, was also studied.

It was suggested that a one-third reduction in oxygen consumption during general anaesthesia could be achieved in the study, involving 20 elderly patients (aged 65 and above) undergoing major abdominal surgery.^3^ In an assessment evaluating the relationship between anaesthesia and oxygen, it was stated that fiO_2 _levels of 30-40% or lower could be sufficient in clinical use if the lungs are kept open. They also mentioned that higher fiO_2 _values might lead to atelectasis.^11^ In our study, the minimum fiO_2 _threshold was established at 30%. If fiO_2_, SpO_2_, or NIRS targets fell below the specified levels, the fgO_2_ was increased by 10%, and changes in oxygen, both in terms of increments and decrements, as well as the total number of alterations, were recorded at the end of each case. When comparing the two groups, there was a statistically significant difference, with a higher number of oxygen increases and alterations in the younger patient group. The lower fgO_2_ levels in the geriatric patient group support our hypothesis, indicating the necessity for lower fgO_2_ levels during MFA to prevent hypoxia. This finding substantiates that MFA can be more readily applied in the geriatric population.

During the intraoperative period, monitoring fiO_2_, SpO_2_, NIRS, or arterial blood gas for PO_2_ and lactate levels is frequently used to ensure adequate oxygenation and avoid hypoxia. Park et al.^12^ conducted a study on 50 patients undergoing laparoscopic robotic surgery lasting more than 6 hours, comparing minimal flow (0.5 L min^-1^,) to the high flow anaesthesia. Although PaO_2_ values were slightly lower in MFA, they remained within reliable limits, demonstrating the safe and effective application of MFA in prolonged laparoscopic surgeries.^12^ The effects of LFA on hemodynamic parameters, recovery time, and arterial blood gas results in morbidly obese patients undergoing laparoscopic sleeve gastrectomy were investigated. The authors found that SpO_2_, arterial blood gas, and recovery times were similar when compared between high-flow anaesthesia and another condition. They suggested that LFA is safe in achieving adequate tissue perfusion, anaesthesia depth, and postoperative recovery in morbidly obese patients.^13^ In our study, although PaO_2_ values were lower in the elderly, they remained within safe limits. Despite a significant decrease in PO_2_ in both groups, the ideal PO_2_ value considered in arterial blood gas measurements, remaining above 100 mmHg, was maintained during the study. SaO_2_ values were maintained above 95% in all patients. Additionally, there was no statistically significant difference in lactate levels. This indicates that MFA can provide adequate tissue oxygenation across different age groups.

The brain is highly susceptible to hypoxia, which may occur during MFA. NIRS is frequently utilized for intraoperative demonstration of cerebral oxygenation and perfusion, and its monitoring has been reported to mitigate postoperative cognitive dysfunction.^14^ The impact of MFA on rSO_2_ and hemodynamics was investigated in the prone position. The study revealed that MFA was associated with higher MAP and left-sided rSO_2_ values compared with normal flow anaesthesia, whereas no significant difference was observed in other vital signs or right-sided rSO_2_. Consequently, their findings suggest that MFA can be considered safe concerning rSO_2_ levels and hemodynamics.^15^ In our study, despite the occasional presence of an intergroup difference in the rSO_2 _values, neither group showed a decrease compared with the baseline value. No significant difference was observed in intragroup comparisons in MAP after switching to MFA, indicating that this technique did not affect cerebral perfusion. This shows that MFA can be performed with close follow-up and appropriate monitoring while preserving cerebral oxygenation and perfusion, in geriatric patients as well.

Awareness under anaesthesia is one of the most undesirable complications during surgery. Monitoring the depth of anaesthesia using vital signs and clinical data can be subjective and insufficiently reliable owing to the presence of numerous factors that may influence these measurements. In the study on awareness under anaesthesia, the incidence of awareness was found to be 1%.^16^ BIS monitoring is often used to prevent this and to provide objective data. The effects of BIS use on awareness in surgeries requiring muscle relaxants and intubation were examined. It was reported that BIS monitoring significantly reduced the incidence of awareness.^17^ In our study, BIS values were maintained at 40-50, which is known as the safe range, in both groups. While no significant intergroup difference was noted in the amount of change in desflurane, the BIS target was maintained within the determined safe range, indicating that MFA can be administered safely in both groups. However, etDes was lower in the older patient group at all times, indicating that adequate depth of anaesthesia was maintained using a reduced dosage of inhalation anaesthetics,  in parallel the risk of inadequate depth of anaesthesia when using MFA was also lower in these patients. No patient described awareness under anaesthesia in the interviews at the 24^th^ postoperative hour.

In normal physiological conditions during anaesthesia, the estimated O_2_ requirement is around 2-3 mL kg^-1 ^min^-1^. Based on this estimation, FGF within the range of 250-500 mL can be safely employed in healthy adults weighing less than 100 kg.^2^ In a study investigating the relationship between LFA and body weight, it was argued that O_2_ requirement should be determined individually in patients under anaesthesia. They stated that the hemodynamic and oxygenation parameters of the patients were maintained within the safe range; thus, FGF-which is determined according to body weight-is more reliable and physiological.^18^ A positive correlation was observed between body weight, BMI, and BSA, and the amount of O_2_ change in all patients in our study, and in both groups. This supported the view that there is a close relationship between body size and O_2 _consumption, which is in line with the previous study. Based on these data, in MFA applications, determining FGF and the O_2 _percentage is thought to be an appropriate approach after determining the O_2_ demand specific to each patient, including elderly patients.

### Study Limitations

This study has several limitations. Firstly, owing to the study’s inherent design, blinding and randomization procedures were not feasible. Secondly, our investigation utilized anaesthesia equipment capable of administering MFA, with 350 mL. This equipment was available solely in two specific operating rooms. Consequently, surgical cases conducted in these two designated rooms were included, resulting in a relatively limited intergroup variance in mean age. Despite this constraint, the outcomes from our study might indicate a more pronounced distinction between younger adults and older patients, necessitating further research for a comprehensive understanding of this topic. It should be noted that patients with severe respiratory and cardiac diseases, which are common comorbidities in the elderly, were excluded from the study. Individual anaesthesia settings for those patients should be considered.

## Conclusion

To the best of our knowledge, this is the first study of comparing the impacts of MFA across various age demographics. Our findings indicate that administering MFA at a rate of 350 mL min^-1^ in the growing cohort of older adult patients can be performed without inducing hypoxia or inadvertent awareness. The use of MFA techniques has proven beneficial in diminishing gas consumption and maintaining adequate oxygen levels with reduced intervention in the elderly population. The incorporation of MFA as an anaesthesia modality should be promoted for multiple reasons: it minimizes respiratory complications when coupled with diligent monitoring and oversight by appropriately trained personnel, aligns with the goal of a sustainable future by contributing less to global warming, and contributes to reducing ambient air pollution within the operating room, benefiting healthcare professionals.

## Ethics

**Ethics Committee Approval:** This prospective study was conducted following approval from the Eskişehir Osmangazi University, Clinical Trials Ethics Committee of Studies (approval no.: 21, dated: December 22, 2022).

**Informed Consent:** Informed consent from all patients was obtained.

## Figures and Tables

**Table 1. Patients’ Demographic Data [Mean ± Standard Deviation, Min.-Max. (Median), Count]:** 

-	**Group Y (n = 40)**	**Group E (n = 40)**	***P* value**
Age	43.9 [25-50 (46)]	70.3 [65-82 (70)]	-
Weight (kg)	77.9±17	74.5±11.5	0.47
BSA (m^2^)	1.9±0.24	1.8±0.16	0.26
BMI (kg m^2-1^)	26.4±4.7	26.1±3.74	0.93
Gender (F/M)	18/22	14/26	0.49
ASA (1/2/3)	3/36/1	2/34/4	0.48

Min.-Max., minimum-maximum; BSA, body surface area; BMI, body mass index; F/M, female/male; ASA, American Society of Anesthesiologists

**Table 2. The Number of Interventions in Intraoperative fgO2 and fgDes Concentrations (Mean ± SD):** 

-	**Group Y**	**Group E**	***P* value**
Number of increases in oxygen	1.8±1.2	1.1±0.8	0.006*
Min.-Max. (median)	0-4 (2)	0-3 (1)	-
Number of decreases in oxygen	0.07±0.26	0.10±0.3	0.9
Min.-Max. (median)	0-1 (0)	0-2 (0)	-
Total alteration in oxygen	1.9±1.3	1.2±1	0.01*
Min.-Max. (median)	0-5 (2)	0-4 (1)	-
Number of increases in desflurane	0.12±0.3	0.07±0.2	0.46
Min.-Max. (median)	0-1 (0)	0-1 (0)	-
Number of decreases in desflurane	4.2±1.7	4.1±1.3	0.47
Min.-Max. (median)	0-7 (4)	2-7 (4)	-
Total alteration in desflurane	4.3±1.8	4.2±1.3	0.46
Min.-Max. (median)	0-8 (5)	2-7 (4)	-

*There is a significant difference between groups (*P *< 0.05)

Min.-Max., minimum-maximum; SD, standard deviation;fgDes, fresh gas desflurane concentration; fgO_2_, fresh gas oxygen concentration

**Table 3. Regional Cerebral Oxygen Saturations (rSO2-R and rSO2-L) (Mean ± Standard Deviation):** 

-	**rSO_2_-R**			**rSO_2_-L**		
-	**Group Y**	**Group E**	***P* value**	**Group Y**	**Group E**	***P* value**
t0	65.6±9.7	67±6.1	0.45	65.2±10.6	66.3±7	0.57
t5	77.6±11.2	75.2±8.2	0.28	76.6±11.4	74.8±7	0.39
t10	76.4±11.1	72.8±9.1	0.11	74.4±11	71.9±7.4	0.25
t15	74±11.7	71±9	0.19	73.2±11.4	70±7.5	0.14
t20	73±11.5	71±8.7	0.38	71.8±10.7	69.7±8	0.32
t25	72.2±11.2	70.4±8.3	0.41	70.9±10.6	68.5±8.1	0.27
t30	72.8±10.7	69.6±8.6	0.15	70.6±10	68.8±7.9	0.37
t40	72.6±10.7	68.9±8	0.08	71.1±10.7	68.3±6.3	0.16
t50	71.8±10	68±7.7	0.05	71±10	67.3±6.8	0.06
t60	73.2±10.7	68.1±7.4	0.01*	71.6±10.2	66.7±6.5	0.01*
t70	71.7±11.4	68.1±7.4	0.09	70.8±10.3	67.3±6.4	0.07
t80	72±11.6	66.9±7.6	0.02*	71.8±11.3	66.4±6.7	0.01*
t90	72.9±10.8	66.4±7.5	0.003*	72.1±11.3	66.2±6.1	0.005*
t100	72.8±10.6	66.9±7	0.004*	72±10.4	66.5±6.2	0.006*
t110	72.6±11.2	67.3±7.3	0.01*	72.5±11.7	66.5±6.5	0.006*
t120	73.4±10.9	67.4±7.4	0.005*	73.4±11.3	66.3±7	0.001*

*There is a significant difference between groups (*P *< 0.05)

rSO_2_-R, regional cerebral oxygen saturation on the right; rSO_2_-L, regional cerebral oxygen saturation on the left

**Table 4. Intraoperative Arterial Blood Gas Measurements (Mean ± Standard Deviation):** 

-	-	**Group Y**	**Group E**	***P* values**
pH	t10	7.38±0.4	7.40±0.4	0.76
	t70	7.36±0.4	7.38±0.4	0.12
PO_2_	t10	183.7±74.4	183.3±88.6	0.9
	t70	117.3±31	106.3±19.6	0.06
PCO_2_	t10	37.7±5.6	36.8±4	0.37
	t70	36.1±4.3	35.6±3.5	0.53
Lactate	t10	1.15±0.56	1.09±0.67	0.67
	t70	1.35±0.62	1.24±0.5	0.40
HCO_3_	t10	22.5±2	22.8±2.2	0.49
	t70	20.8±2.1	21.3±2.2	0.24

There are no significant differences between the groups (*P *> 0.05)

**Table 5. Comparison of Desflurane, O2, Air and Remifentanil Consumption Between Groups:** 

-	**Group Y**	**Group E**	***P *value**
Desflurane consumption	38.2±7.1	34.3±5.3	0.012*
O_2_ consumption	125.4±27.9	127.2±30.4	0.92
Air consumption	40.2±6.3	43±10.1	0.25
Remifentanil consumption (mL)	36.4±7.8	34.9±6.4	0.4

*There is a significant difference between groups (*P*****< 0.05)
